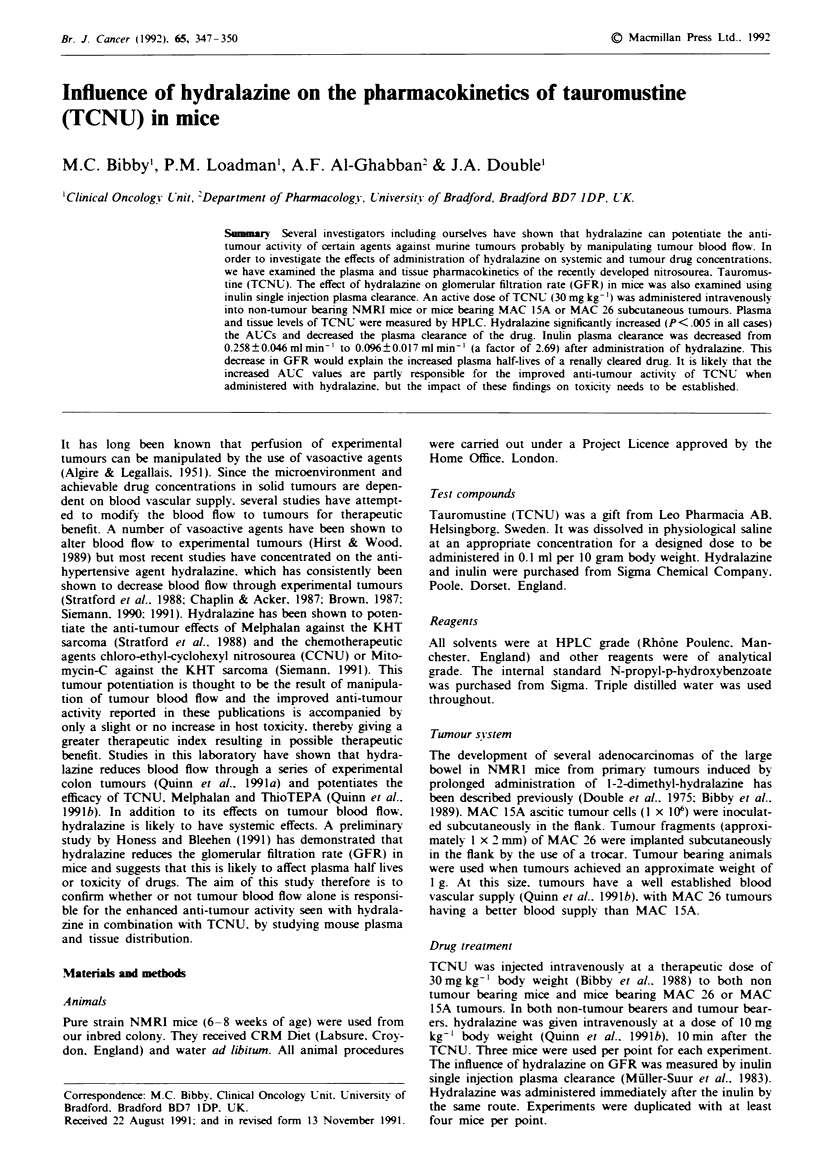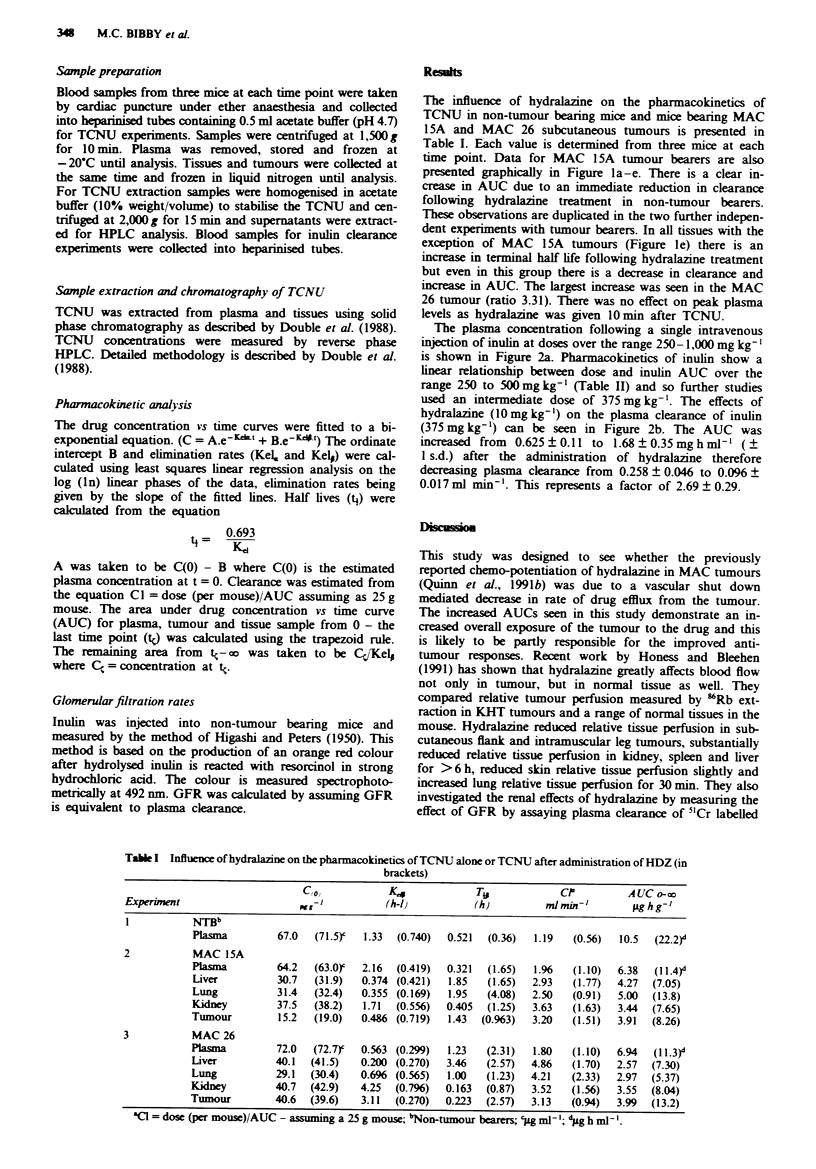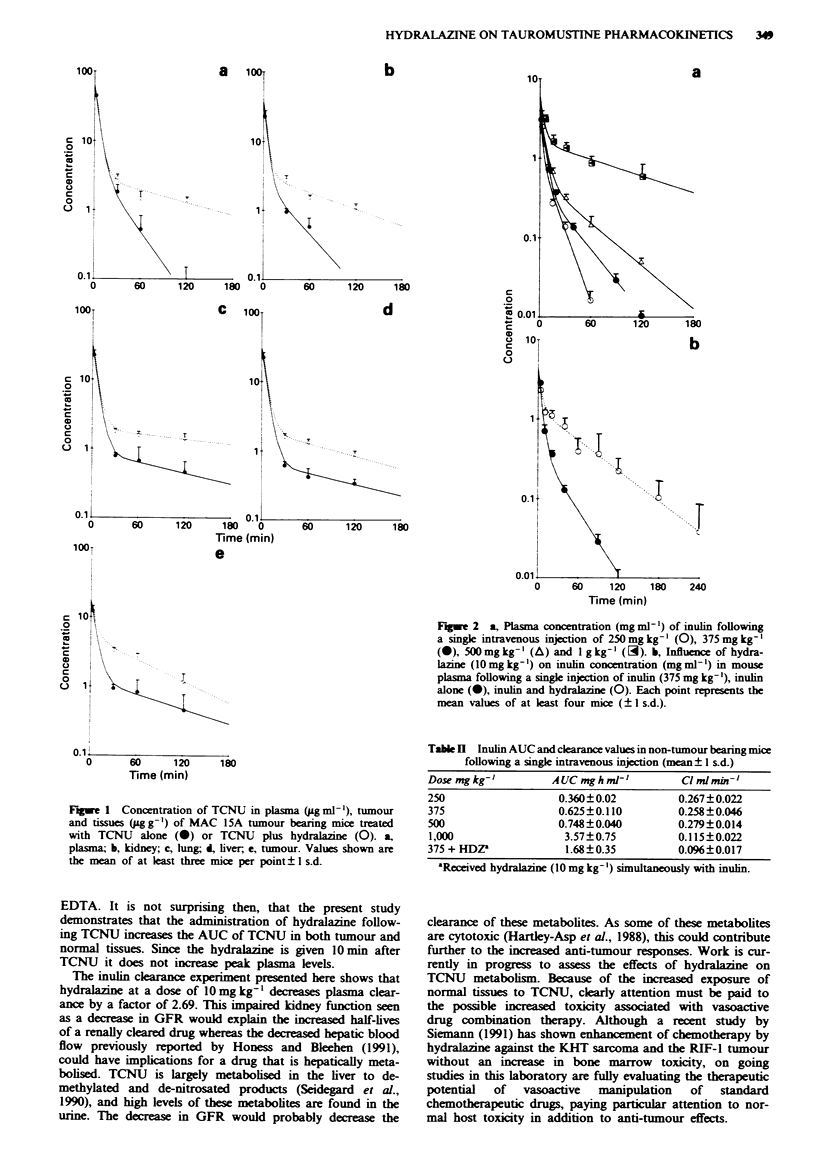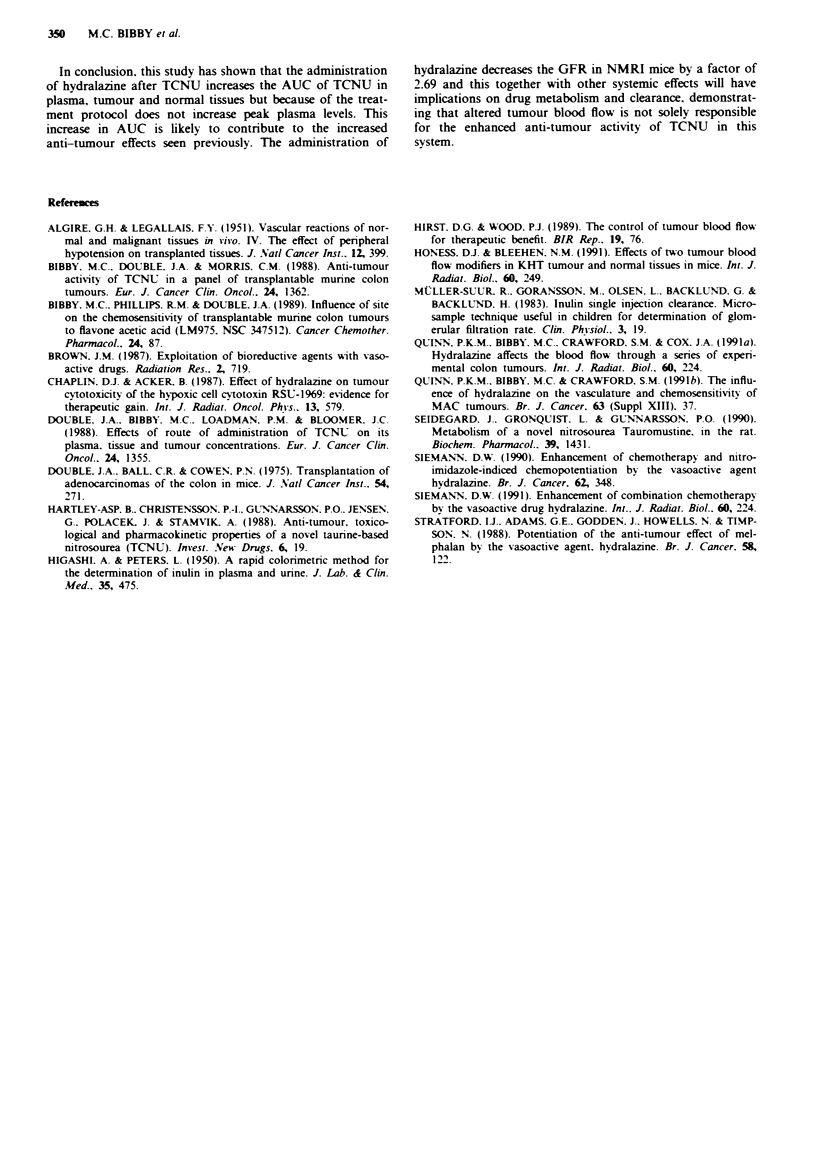# Influence of hydralazine on the pharmacokinetics of tauromustine (TCNU) in mice.

**DOI:** 10.1038/bjc.1992.70

**Published:** 1992-03

**Authors:** M. C. Bibby, P. M. Loadman, A. F. al-Ghabban, J. A. Double

**Affiliations:** Clinical Oncology Unit, University of Bradford, UK.

## Abstract

Several investigators including ourselves have shown that hydralazine can potentiate the anti-tumour activity of certain agents against murine tumours probably by manipulating tumour blood flow. In order to investigate the effects of administration of hydralazine on systemic and tumour drug concentrations, we have examined the plasma and tissue pharmacokinetics of the recently developed nitrosourea, Tauromustine (TCNU). The effect of hydralazine on glomerular filtration rate (GFR) in mice was also examined using inulin single injection plasma clearance. An active dose of TCNU (30 mg kg-1) was administered intravenously into non-tumour bearing NMRI mice or mice bearing MAC 15A or MAC 26 subcutaneous tumours. Plasma and tissue levels of TCNU were measured by HPLC. Hydralazine significantly increased (P less than .005 in all cases) the AUCs and decreased the plasma clearance of the drug. Inulin plasma clearance was decreased from 0.258 +/- 0.046 ml min-1 to 0.096 +/- 0.017 ml min-1 (a factor of 2.69) after administration of hydralazine. This decrease in GFR would explain the increased plasma half-lives of a renally cleared drug. It is likely that the increased AUC values are partly responsible for the improved anti-tumour activity of TCNU when administered with hydralazine, but the impact of these findings on toxicity needs to be established.


Br. J. Cancer (1992). 65, 347-350 © Macmillan Press Ltd., 1992

### Influence of hydralazine on the pharmacokinetics of tauromustine (TCNU) in mice

M.C. Bibby[1], P.M. Loadman[1], A.F. Al-Ghabban[2] & J.A. Double[1]

[1]Clinical Oncology Unit, [2]Department of Pharmacology, University of Bradford, Bradford BD7 1DP, UK.

**Summary** Several investigators including ourselves have shown that hydralazine can potentiate the anti-tumour activity of certain agents against murine tumours probably by manipulating tumour blood flow. In order to investigate the effects of administration of hydralazine on systemic and tumour drug concentrations, we have examined the plasma and tissue pharmacokinetics of the recently developed nitrosourea. Tauromustine (TCNU). The effect of hydralazine on glomerular filtration rate (GFR) in mice was also examined using inulin single injection plasma clearance. An active dose of TCNU (30 mg $kg^{-1}$) was administered intravenously into non-tumour bearing NMRI mice or mice bearing MAC 15A or MAC 26 subcutaneous tumours. Plasma and tissue levels of TCNU were measured by HPLC. Hydralazine significantly increased ($P < .005$ in all cases) the AUCs and decreased the plasma clearance of the drug. Inulin plasma clearance was decreased from $0.258 \pm 0.046$ ml $min^{-1}$ to $0.096 \pm 0.017$ ml $min^{-1}$ (a factor of 2.69) after administration of hydralazine. This decrease in GFR would explain the increased plasma half-lives of a renally cleared drug. It is likely that the increased AUC values are partly responsible for the improved anti-tumour activity of TCNU when administered with hydralazine, but the impact of these findings on toxicity needs to be established.

It has long been known that perfusion of experimental tumours can be manipulated by the use of vasoactive agents (Algire & Legallais, 1951). Since the microenvironment and achievable drug concentrations in solid tumours are dependent on blood vascular supply, several studies have attempted to modify the blood flow to tumours for therapeutic benefit. A number of vasoactive agents have been shown to alter blood flow to experimental tumours (Hirst & Wood, 1989) but most recent studies have concentrated on the anti-hypertensive agent hydralazine, which has consistently been shown to decrease blood flow through experimental tumours (Stratford *et al.*, 1988; Chaplin & Acker, 1987; Brown, 1987; Siemann, 1990; 1991). Hydralazine has been shown to potentiate the anti-tumour effects of Melphalan against the KHT sarcoma (Stratford *et al.*, 1988) and the chemotherapeutic agents chloro-ethyl-cyclohexyl nitrosourea (CCNU) or Mitomycin-C against the KHT sarcoma (Siemann, 1991). This tumour potentiation is thought to be the result of manipulation of tumour blood flow and the improved anti-tumour activity reported in these publications is accompanied by only a slight or no increase in host toxicity, thereby giving a greater therapeutic index resulting in possible therapeutic benefit. Studies in this laboratory have shown that hydralazine reduces blood flow through a series of experimental colon tumours (Quinn *et al.*, 1991*a*) and potentiates the efficacy of TCNU, Melphalan and ThioTEPA (Quinn *et al.*, 1991*b*). In addition to its effects on tumour blood flow, hydralazine is likely to have systemic effects. A preliminary study by Honess and Bleehen (1991) has demonstrated that hydralazine reduces the glomerular filtration rate (GFR) in mice and suggests that this is likely to affect plasma half lives or toxicity of drugs. The aim of this study therefore is to confirm whether or not tumour blood flow alone is responsible for the enhanced anti-tumour activity seen with hydralazine in combination with TCNU, by studying mouse plasma and tissue distribution.

### Materials and methods

### *Animals*

Pure strain NMRI mice (6-8 weeks of age) were used from our inbred colony. They received CRM Diet (Labsure, Croydon, England) and water *ad libitum*. All animal procedures were carried out under a Project Licence approved by the Home Office, London.

### *Test compounds*

Tauromustine (TCNU) was a gift from Leo Pharmacia AB, Helsingborg, Sweden. It was dissolved in physiological saline at an appropriate concentration for a designed dose to be administered in 0.1 ml per 10 gram body weight. Hydralazine and inulin were purchased from Sigma Chemical Company, Poole, Dorset, England.

### *Reagents*

All solvents were at HPLC grade (Rhône Poulenc, Manchester, England) and other reagents were of analytical grade. The internal standard N-propyl-p-hydroxybenzoate was purchased from Sigma. Triple distilled water was used throughout.

### *Tumour system*

The development of several adenocarcinomas of the large bowel in NMRI mice from primary tumours induced by prolonged administration of 1-2-dimethyl-hydralazine has been described previously (Double *et al.*, 1975; Bibby *et al.*, 1989). MAC 15A ascitic tumour cells ($1 \times 10^6$) were inoculated subcutaneously in the flank. Tumour fragments (approximately $1 \times 2$ mm) of MAC 26 were implanted subcutaneously in the flank by the use of a trocar. Tumour bearing animals were used when tumours achieved an approximate weight of 1 g. At this size, tumours have a well established blood vascular supply (Quinn *et al.*, 1991*b*), with MAC 26 tumours having a better blood supply than MAC 15A.

### *Drug treatment*

TCNU was injected intravenously at a therapeutic dose of 30 mg $kg^{-1}$ body weight (Bibby *et al.*, 1988) to both non tumour bearing mice and mice bearing MAC 26 or MAC 15A tumours. In both non-tumour bearers and tumour bearers, hydralazine was given intravenously at a dose of 10 mg $kg^{-1}$ body weight (Quinn *et al.*, 1991*b*), 10 min after the TCNU. Three mice were used per point for each experiment. The influence of hydralazine on GFR was measured by inulin single injection plasma clearance (Müller-Suur *et al.*, 1983). Hydralazine was administered immediately after the inulin by the same route. Experiments were duplicated with at least four mice per point.

Correspondence: M.C. Bibby, Clinical Oncology Unit, University of Bradford, Bradford BD7 1DP, UK.
Received 22 August 1991; and in revised form 13 November 1991.

### Sample preparation

Blood samples from three mice at each time point were taken by cardiac puncture under ether anaesthesia and collected into heparinised tubes containing 0.5 ml acetate buffer (pH 4.7) for TCNU experiments. Samples were centrifuged at 1,500 *g* for 10 min. Plasma was removed, stored and frozen at −20°C until analysis. Tissues and tumours were collected at the same time and frozen in liquid nitrogen until analysis. For TCNU extraction samples were homogenised in acetate buffer (10% weight/volume) to stabilise the TCNU and centrifuged at 2,000 *g* for 15 min and supernatants were extracted for HPLC analysis. Blood samples for inulin clearance experiments were collected into heparinised tubes.

### Sample extraction and chromatography of TCNU

TCNU was extracted from plasma and tissues using solid phase chromatography as described by Double *et al.* (1988). TCNU concentrations were measured by reverse phase HPLC. Detailed methodology is described by Double *et al.* (1988).

### Pharmacokinetic analysis

The drug concentration *vs* time curves were fitted to a bi-exponential equation. ($C = A.e^{-Kel_{\alpha}.t} + B.e^{-Kel_{\beta}.t}$) The ordinate intercept B and elimination rates ($Kel_{\alpha}$ and $Kel_{\beta}$) were calculated using least squares linear regression analysis on the log (1n) linear phases of the data, elimination rates being given by the slope of the fitted lines. Half lives ($t_{\frac{1}{2}}$) were calculated from the equation

$$t_{\frac{1}{2}} = \frac{0.693}{K_{el}}$$

A was taken to be C(0) − B where C(0) is the estimated plasma concentration at t = 0. Clearance was estimated from the equation Cl = dose (per mouse)/AUC assuming as 25 g mouse. The area under drug concentration *vs* time curve (AUC) for plasma, tumour and tissue sample from 0 − the last time point ($t_{\zeta}$) was calculated using the trapezoid rule. The remaining area from $t_{\zeta} - \infty$ was taken to be $C_{\zeta}/Kel_{\beta}$ where $C_{\zeta}$ = concentration at $t_{\zeta}$.

### Glomerular filtration rates

Inulin was injected into non-tumour bearing mice and measured by the method of Higashi and Peters (1950). This method is based on the production of an orange red colour after hydrolysed inulin is reacted with resorcinol in strong hydrochloric acid. The colour is measured spectrophotometrically at 492 nm. GFR was calculated by assuming GFR is equivalent to plasma clearance.

### Results

The influence of hydralazine on the pharmacokinetics of TCNU in non-tumour bearing mice and mice bearing MAC 15A and MAC 26 subcutaneous tumours is presented in Table I. Each value is determined from three mice at each time point. Data for MAC 15A tumour bearers are also presented graphically in Figure 1a–e. There is a clear increase in AUC due to an immediate reduction in clearance following hydralazine treatment in non-tumour bearers. These observations are duplicated in the two further independent experiments with tumour bearers. In all tissues with the exception of MAC 15A tumours (Figure 1e) there is an increase in terminal half life following hydralazine treatment but even in this group there is a decrease in clearance and increase in AUC. The largest increase was seen in the MAC 26 tumour (ratio 3.31). There was no effect on peak plasma levels as hydralazine was given 10 min after TCNU.

The plasma concentration following a single intravenous injection of inulin at doses over the range 250–1,000 mg $kg^{-1}$ is shown in Figure 2a. Pharmacokinetics of inulin show a linear relationship between dose and inulin AUC over the range 250 to 500 mg $kg^{-1}$ (Table II) and so further studies used an intermediate dose of 375 mg $kg^{-1}$. The effects of hydralazine (10 mg $kg^{-1}$) on the plasma clearance of inulin (375 mg $kg^{-1}$) can be seen in Figure 2b. The AUC was increased from $0.625 \pm 0.11$ to $1.68 \pm 0.35$ mg h $ml^{-1}$ (± 1 s.d.) after the administration of hydralazine therefore decreasing plasma clearance from $0.258 \pm 0.046$ to $0.096 \pm 0.017$ ml $min^{-1}$. This represents a factor of $2.69 \pm 0.29$.

### Discussion

This study was designed to see whether the previously reported chemo-potentiation of hydralazine in MAC tumours (Quinn *et al.*, 1991*b*) was due to a vascular shut down mediated decrease in rate of drug efflux from the tumour. The increased AUCs seen in this study demonstrate an increased overall exposure of the tumour to the drug and this is likely to be partly responsible for the improved anti-tumour responses. Recent work by Honess and Bleehen (1991) has shown that hydralazine greatly affects blood flow not only in tumour, but in normal tissue as well. They compared relative tumour perfusion measured by $^{86}Rb$ extraction in KHT tumours and a range of normal tissues in the mouse. Hydralazine reduced relative tissue perfusion in subcutaneous flank and intramuscular leg tumours, substantially reduced relative tissue perfusion in kidney, spleen and liver for >6 h, reduced skin relative tissue perfusion slightly and increased lung relative tissue perfusion for 30 min. They also investigated the renal effects of hydralazine by measuring the effect of GFR by assaying plasma clearance of $^{51}Cr$ labelled

**Table I** Influence of hydralazine on the pharmacokinetics of TCNU alone or TCNU after administration of HDZ (in brackets)

| Experiment | | $C_{(0)}$ µg $g^{-1}$ | | $K_{el\beta}$ (h-1) | | $T_{\frac{1}{2}\beta}$ (h) | | Cl[a] ml $min^{-1}$ | | AUC 0-∞ µg h $g^{-1}$ | |
|---|---|---|---|---|---|---|---|---|---|---|---|
| 1 | NTB[b] | | | | | | | | | | |
| | Plasma | 67.0 | (71.5)[c] | 1.33 | (0.740) | 0.521 | (0.36) | 1.19 | (0.56) | 10.5 | (22.2)[d] |
| 2 | MAC 15A | | | | | | | | | | |
| | Plasma | 64.2 | (63.0)[c] | 2.16 | (0.419) | 0.321 | (1.65) | 1.96 | (1.10) | 6.38 | (11.4)[d] |
| | Liver | 30.7 | (31.9) | 0.374 | (0.421) | 1.85 | (1.65) | 2.93 | (1.77) | 4.27 | (7.05) |
| | Lung | 31.4 | (32.4) | 0.355 | (0.169) | 1.95 | (4.08) | 2.50 | (0.91) | 5.00 | (13.8) |
| | Kidney | 37.5 | (38.2) | 1.71 | (0.556) | 0.405 | (1.25) | 3.63 | (1.63) | 3.44 | (7.65) |
| | Tumour | 15.2 | (19.0) | 0.486 | (0.719) | 1.43 | (0.963) | 3.20 | (1.51) | 3.91 | (8.26) |
| 3 | MAC 26 | | | | | | | | | | |
| | Plasma | 72.0 | (72.7)[c] | 0.563 | (0.299) | 1.23 | (2.31) | 1.80 | (1.10) | 6.94 | (11.3)[d] |
| | Liver | 40.1 | (41.5) | 0.200 | (0.270) | 3.46 | (2.57) | 4.86 | (1.70) | 2.57 | (7.30) |
| | Lung | 29.1 | (30.4) | 0.696 | (0.565) | 1.00 | (1.23) | 4.21 | (2.33) | 2.97 | (5.37) |
| | Kidney | 40.7 | (42.9) | 4.25 | (0.796) | 0.163 | (0.87) | 3.52 | (1.56) | 3.55 | (8.04) |
| | Tumour | 40.6 | (39.6) | 3.11 | (0.270) | 0.223 | (2.57) | 3.13 | (0.94) | 3.99 | (13.2) |

[a]Cl = dose (per mouse)/AUC – assuming a 25 g mouse; [b]Non-tumour bearers; [c]µg $ml^{-1}$; [d]µg h $ml^{-1}$.

**Figure 1** Concentration of TCNU in plasma ($\mu g\ ml^{-1}$), tumour and tissues ($\mu g\ g^{-1}$) of MAC 15A tumour bearing mice treated with TCNU alone (●) or TCNU plus hydralazine (O). **a**, plasma; **b**, kidney; **c**, lung; **d**, liver; **e**, tumour. Values shown are the mean of at least three mice per point ± 1 s.d.

**Figure 2** **a**, Plasma concentration ($mg\ ml^{-1}$) of inulin following a single intravenous injection of 250 mg $kg^{-1}$ (O), 375 mg $kg^{-1}$ (●), 500 mg $kg^{-1}$ (Δ) and 1 g $kg^{-1}$ (◪). **b**, Influence of hydralazine (10 mg $kg^{-1}$) on inulin concentration ($mg\ ml^{-1}$) in mouse plasma following a single injection of inulin (375 mg $kg^{-1}$), inulin alone (●), inulin and hydralazine (O). Each point represents the mean values of at least four mice (± 1 s.d.).

**Table II** Inulin AUC and clearance values in non-tumour bearing mice following a single intravenous injection (mean ± 1 s.d.)

| *Dose mg kg$^{-1}$* | *AUC mg h ml$^{-1}$* | *Cl ml min$^{-1}$* |
|---|---|---|
| 250 | 0.360 ± 0.02 | 0.267 ± 0.022 |
| 375 | 0.625 ± 0.110 | 0.258 ± 0.046 |
| 500 | 0.748 ± 0.040 | 0.279 ± 0.014 |
| 1,000 | 3.57 ± 0.75 | 0.115 ± 0.022 |
| 375 + HDZ[a] | 1.68 ± 0.35 | 0.096 ± 0.017 |

[a]Received hydralazine (10 mg $kg^{-1}$) simultaneously with inulin.

EDTA. It is not surprising then, that the present study demonstrates that the administration of hydralazine following TCNU increases the AUC of TCNU in both tumour and normal tissues. Since the hydralazine is given 10 min after TCNU it does not increase peak plasma levels.

The inulin clearance experiment presented here shows that hydralazine at a dose of 10 mg $kg^{-1}$ decreases plasma clearance by a factor of 2.69. This impaired kidney function seen as a decrease in GFR would explain the increased half-lives of a renally cleared drug whereas the decreased hepatic blood flow previously reported by Honess and Bleehen (1991), could have implications for a drug that is hepatically metabolised. TCNU is largely metabolised in the liver to demethylated and de-nitrosated products (Seidegard *et al.*, 1990), and high levels of these metabolites are found in the urine. The decrease in GFR would probably decrease the clearance of these metabolites. As some of these metabolites are cytotoxic (Hartley-Asp *et al.*, 1988), this could contribute further to the increased anti-tumour responses. Work is currently in progress to assess the effects of hydralazine on TCNU metabolism. Because of the increased exposure of normal tissues to TCNU, clearly attention must be paid to the possible increased toxicity associated with vasoactive drug combination therapy. Although a recent study by Siemann (1991) has shown enhancement of chemotherapy by hydralazine against the KHT sarcoma and the RIF-1 tumour without an increase in bone marrow toxicity, on going studies in this laboratory are fully evaluating the therapeutic potential of vasoactive manipulation of standard chemotherapeutic drugs, paying particular attention to normal host toxicity in addition to anti-tumour effects.

In conclusion, this study has shown that the administration of hydralazine after TCNU increases the AUC of TCNU in plasma, tumour and normal tissues but because of the treatment protocol does not increase peak plasma levels. This increase in AUC is likely to contribute to the increased anti–tumour effects seen previously. The administration of hydralazine decreases the GFR in NMRI mice by a factor of 2.69 and this together with other systemic effects will have implications on drug metabolism and clearance, demonstrating that altered tumour blood flow is not solely responsible for the enhanced anti-tumour activity of TCNU in this system.

### References

ALGIRE, G.H. & LEGALLAIS, F.Y. (1951). Vascular reactions of normal and malignant tissues *in vivo*. IV. The effect of peripheral hypotension on transplanted tissues. *J. Natl Cancer Inst.*, **12**, 399.

BIBBY, M.C., DOUBLE, J.A. & MORRIS, C.M. (1988). Anti-tumour activity of TCNU in a panel of transplantable murine colon tumours. *Eur. J. Cancer Clin. Oncol.*, **24**, 1362.

BIBBY, M.C., PHILLIPS, R.M. & DOUBLE, J.A. (1989). Influence of site on the chemosensitivity of transplantable murine colon tumours to flavone acetic acid (LM975, NSC 347512). *Cancer Chemother. Pharmacol.*, **24**, 87.

BROWN, J.M. (1987). Exploitation of bioreductive agents with vasoactive drugs. *Radiation Res.*, **2**, 719.

CHAPLIN, D.J. & ACKER, B. (1987). Effect of hydralazine on tumour cytotoxicity of the hypoxic cell cytotoxin RSU-1969: evidence for therapeutic gain. *Int. J. Radiat. Oncol. Phys.*, **13**, 579.

DOUBLE, J.A., BIBBY, M.C., LOADMAN, P.M. & BLOOMER, J.C. (1988). Effects of route of administration of TCNU on its plasma, tissue and tumour concentrations. *Eur. J. Cancer Clin. Oncol.*, **24**, 1355.

DOUBLE, J.A., BALL, C.R. & COWEN, P.N. (1975). Transplantation of adenocarcinomas of the colon in mice. *J. Natl Cancer Inst.*, **54**, 271.

HARTLEY-ASP, B., CHRISTENSSON, P.-I., GUNNARSSON, P.O., JENSEN, G., POLACEK, J. & STAMVIK, A. (1988). Anti-tumour, toxicological and pharmacokinetic properties of a novel taurine-based nitrosourea (TCNU). *Invest. New Drugs*, **6**, 19.

HIGASHI, A. & PETERS, L. (1950). A rapid colorimetric method for the determination of inulin in plasma and urine. *J. Lab. & Clin. Med.*, **35**, 475.

HIRST, D.G. & WOOD, P.J. (1989). The control of tumour blood flow for therapeutic benefit. *BIR Rep.*, **19**, 76.

HONESS, D.J. & BLEEHEN, N.M. (1991). Effects of two tumour blood flow modifiers in KHT tumour and normal tissues in mice. *Int. J. Radiat. Biol.*, **60**, 249.

MÜLLER-SUUR, R., GORANSSON, M., OLSEN, L., BACKLUND, G. & BACKLUND, H. (1983). Inulin single injection clearance. Microsample technique useful in children for determination of glomerular filtration rate. *Clin. Physiol.*, **3**, 19.

QUINN, P.K.M., BIBBY, M.C., CRAWFORD, S.M. & COX, J.A. (1991*a*). Hydralazine affects the blood flow through a series of experimental colon tumours. *Int. J. Radiat. Biol.*, **60**, 224.

QUINN, P.K.M., BIBBY, M.C. & CRAWFORD, S.M. (1991*b*). The influence of hydralazine on the vasculature and chemosensitivity of MAC tumours. *Br. J. Cancer*, **63** (Suppl XIII), 37.

SEIDEGARD, J., GRONQUIST, L. & GUNNARSSON, P.O. (1990). Metabolism of a novel nitrosourea Tauromustine, in the rat. *Biochem. Pharmacol.*, **39**, 1431.

SIEMANN, D.W. (1990). Enhancement of chemotherapy and nitroimidazole-indiced chemopotentiation by the vasoactive agent hydralazine. *Br. J. Cancer*, **62**, 348.

SIEMANN, D.W. (1991). Enhancement of combination chemotherapy by the vasoactive drug hydralazine. *Int., J. Radiat. Biol.*, **60**, 224.

STRATFORD, I.J., ADAMS, G.E., GODDEN, J., HOWELLS, N. & TIMPSON, N. (1988). Potentiation of the anti-tumour effect of melphalan by the vasoactive agent, hydralazine. *Br. J. Cancer*, **58**, 122.